# Gene–Air Pollution Interaction in Cardiovascular Disease: Lights and Shadows in a Tangled Risk Factor Network

**DOI:** 10.3390/ijms27156818

**Published:** 2026-07-29

**Authors:** Melania Gaggini, Cristina Vassalle

**Affiliations:** 1 Institute of Clinical Physiology, CNR, 56124 Pisa, Italy; melania.gaggini@cnr.it; 2 Fondazione Toscana Gabriele Monasterio, 56124 Pisa, Italy

**Keywords:** genes, air pollution, cardiovascular disease, acute myocardial infarction

## Abstract

Cardiovascular disease (CVD) is the result of a complex interaction between genetic, lifestyle, and environmental factors, which may vary over the course of life. Traditional risk factors do not explain all patients’ risks; thus, the research of additional biomarkers to refine cardiovascular risk prediction has attracted considerable interest in recent years. Genetic factors, as well as air pollution exposure, among nontraditional factors, have been found to be critical determinants of CV risk. Accordingly, this narrative review aims to provide a comprehensive summary of the literature (PubMed) on the combined effects of air pollution and genetic susceptibility on CVD risk. Although different limitations and pitfalls are still to be solved, available evidence suggests that genetic variants (especially genes related to detoxification and inflammation) are involved in the association between air pollution exposure and CV adverse events. Thus, this research area could provide further knowledge of the etiology of CVD, offering new tools for targeted prevention and treatment of more susceptible subjects from a more personalized medicine point of view.

## 1. Introduction

Cardiovascular disease (CVD) remains a major cause of morbidity and mortality all over the world, a fact that highlights the importance of primary and secondary prevention [1]. In this context, well-known established CVD risk factors are considered to be elevated blood pressure, metabolic risk factors, and inflammation [2]. In particular, biochemical markers, such as cholesterol levels and C reactive protein (CRP), have enhanced our knowledge of cardiovascular risk [3]. However, traditional risk factors fail to identify all patients who are at risk of developing cardiovascular disease or precipitate acute cardiovascular (CV) events. This fact may be due, in part, to the different responses of subjects to similar levels of a specific biomarker, as well as to their fluctuation over time, which may be significant because other factors can modify the impact of these parameters on a subject’s risk. In any case, the use of additional newly proposed biomarkers to increase traditional CV risk prediction has attracted considerable interest in recent years. These additive biomarkers include various categories, including blood biochemical parameters, imaging and genetic markers, metabolomics, and microRNAs [4]. Each of these biomarkers may reflect different biological phases in disease progression; e.g., blood biomarkers may be informative either at early or late stages in disease progression, and in a dynamic way, imaging biomarkers may identify subclinical disease, inflammation, and plaque instability. However, genetic biomarkers provide information on disease susceptibility in a static sense, which implies without indicating disease progression. Utilizing combined biomarkers (especially those of a different nature) may pay off because it may give gain in risk prevention in patients most at risk of developing disease or experiencing adverse events, allowing timely interventions and personalized treatment, as well as reducing the healthcare burden and containing healthcare costs. However, there is an extreme complexity when gathering this information, since CV disease is the result of a tangled interaction between genetic, lifestyle, and environmental factors, which may vary over the course of life [5].

In recent years, both genetic predisposition and air pollution exposure, among nontraditional factors, have been identified as critical determinants of CV risk [6,7,8]. More interestingly, other data have evidenced the combined and mutual effects of air pollution and genetic susceptibility on the onset and progression of cardiometabolic diseases [9,10,11]. Thus, control of air quality may be beneficial for CVD, especially in patients with a particular gene profile conferring a higher risk. Moreover, the integration of this information with traditional risk factors could further improve CV risk prediction.

Accordingly, this narrative review aims to provide a comprehensive summary of the literature found in PubMed on the combined effects of air pollution and genetic susceptibility on CVD risk, thus evidencing the advantages, obstacles to overcome, and the benefits of employing a multimarker approach.

Different combinations of the following main key words were used: air pollution, cardiovascular disease, gene–environment interaction, genetic susceptibility, polygenic risk score, environmental exposure, particulate matter, PM2.5, oxidative stress, inflammation, nitrogen dioxide, and personalized prevention. Research was limited to articles published in English to ensure accurate translation and comprehension. Selection was based on the title, abstract, and full text of each paper, primarily focusing on studies published in the last 5 years. However, some key articles published before that date were also cited to provide contextual background.

As a narrative review, the present manuscript provides a comprehensive and integrative overview of the current evidence in this rapidly evolving field, allowing the synthesis of data from heterogeneous studies and the discussion of emerging mechanistic insights, clinical implications, and areas of ongoing development. However, this methodology has well-known limitations, because, unlike systematic reviews, it does not provide a predefined, reproducible search strategy and may therefore be potentially susceptible to selection and publication bias. Moreover, the estimation of the magnitude of effects or definitive conclusions regarding efficacy or causality cannot be drawn. In conclusion, the present work should be considered as a comprehensive synthesis of the current data rather than as definitive evidence, which underlines the need for further prospective results and systematic reviews and meta-analyses.

## 2. Where We Are

### 2.1. Air Pollution-Related CV Risk

Different meta-analyses have reported consistent evidence on the relationship between exposure to air pollutants (especially PM2.5, which means particulate matter with diameters < 2.5 µm, but not only that) and clinical cardiometabolic diseases, such as an increased risk of myocardial infarction (MI), metabolic syndrome, type 2 diabetes, ischemic stroke, or heart failure, and CV mortality [12,13,14,15,16,17].

Specifically, a systematic review and meta-analysis including 18 prospective studies involving 7,300,591 participants, with a median follow-up of 9 years, investigated the association between PM2.5 exposure and cardiovascular disease, cardiovascular events, and all-cause mortality [12]. The analysis demonstrated that higher exposure to PM2.5 was associated with an increased risk of all-cause mortality (HR 1.08, 95% CI 1.05–1.11; *p* < 0.05), cardiovascular disease (HR 1.09, 95% CI 1.00–1.18; *p* < 0.05), and cardiovascular disease mortality (HR 1.12, 95% CI 1.07–1.18; *p* < 0.05) [12]. Moreover, a recent meta-analysis also evidenced the association between PM10 exposure and the risk of MI [13].

Another systematic review and meta-analysis (111 articles with >65 million participants) reported an association between short-term exposure to ozone [O_3_] pollution and the risk of hospital admission for an MI or stroke [18]. These data were confirmed by another systematic review/meta-analysis (18,035,408 cases of ischemic stroke from 110 observational studies), which found significant correlations between short-term exposure to gaseous and particulate air pollutants (carbon monoxide [CO], sulfur dioxide [SO_2_], nitrogen dioxide [NO_2_], O_3_, PM2.5, and PM10), and the occurrence and mortality rates of stroke as follows: stroke incidence was associated with an increase in levels of NO_2_, O_3_, CO, SO_2_, PM2.5, and PM10, whereas stroke mortality was associated with an increase in NO_2_, SO_2_, PM2.5, and PM10 [19].

Moreover, a relationship between short- and long-term air pollution exposure (PM2.5, PM10, NO_2_, SO_2_, CO, and O_3_) and heart failure (HF) hospitalization, incidence, and mortality was evaluated in a meta-analysis (81 short-term studies and 19 studies on long-term exposure), the results of which evidenced an adverse association between air pollution and HF [17].

The analysis of twelve studies (663,276 angina events from Asia, America, Oceania, and Europe) reported that each 10 µg/m^3^ increase in PM2.5, PM10, NO_2_, and CO was associated with an increase in the risk of developing angina, with elderly and patients with coronary artery disease (CAD) at higher risk of an adverse event [20]. Another recent meta-analysis (17 studies; 28,186,905 cases) evidenced that high concentrations of air pollutants (those evaluated were PM2.5, PM10, NO_2_, SO_2_, CO, and O_3_) may significantly contribute to an increased risk of hospitalization due to ischemic heart disease (PM2.5: [RR = 1.01 (95% confidence interval (CI):1.00~1.01), *p* = 0.000]; PM10: [RR = 1.01 (95%CI:1.00~1.01), *p* = 0.000]; NO_2_: [RR = 1.02 (95%CI: 1.00~1.03), *p* = 0.000]; SO_2_: [RR = 1.01 (95%CI: 1.00~1.02), *p* = 0.001]; CO: [RR = 1.04 (95%CI: 0.97~1.12), *p* = 0.01]; and O_3_: [RR = 1.00 (95%CI: 1.00~1.00), *p* = 0.000]) [21].

The association between particulate and gaseous pollutants (including PM10, PM2.5, NO_2_, SO_2_, O_3_, and CO) and CV morbidity and mortality was also confirmed in low-and middle-income countries, where a relationship was observed for other environment-related parameters, such as high and low temperatures and long-term solid fuel exposure, with CV deaths [22]. Moreover, a systematic review and meta-analysis (six cohort studies; 11,656 participants) evidenced significant associations between NO_2_ and PM10 (but not PM2.5) with insulin resistance biomarkers [23]. The results from these studies are summarized in Table 1.

Thus, altogether, the available data agree that air pollution may represent an important modifiable cardiometabolic risk factor, which merits being integrated into clinical CV management instead of being neglected in clinical practice. 

The main mechanisms linking air pollution and CVD have been proposed, with the main culprits identified being oxidative stress and inflammation, to which other adverse effects are ascribable (e.g., endothelial dysfunction, atherosclerosis, autonomic nervous system imbalance, as well as increased coagulation, thrombosis, and blood pressure, and cardiac dysfunction by promotion of cardiac arrhythmias and altered heart rate variability) [24]. In this context, PM2.5 is considered particularly harmful because of its small particle size and greater ability to pass through the alveolar barrier, and as such, has been the most extensively studied. PM2.5 causes inflammation, oxidative stress, and vascular dysfunction directly or indirectly (through particles, metals, and other components), which, in turn, promote the onset and development of atherosclerotic lesions until clinical manifestations of CVD occur [25,26].

### 2.2. Gene-Related CV Risk

In recent years, increasingly larger genome-wide association studies (GWAS) have demonstrated how thousands of genetic loci are associated with cardiometabolic diseases (more than 300 loci with CAD, and more than 1000 associated with CV risk factors) and CAD outcomes, even after adjustments for traditional risk factors. Indeed, every single variant accounts for a small risk, but together, they may be responsible for a considerable cardiovascular risk burden [27,28,29]. Although genetic information from a single/few genetic variant/s is restricted to a generally limited effect, these results allowed the aggregation of significant variants associated with a particular disease to create a polygenic risk score (PRS), providing an overall genetic risk, representing the weighted sum of these variants derived from genome-wide association studies. In this context, PRS may help to refine predictive capacity, better identifying subjects at an increased risk of developing cardiometabolic conditions [30]. Thus, PRS may enhance the predictive accuracy of established clinical scores, increasing the accuracy of an individual’s risk prediction [31]. In fact, the inclusion of polygenic risk beyond conventional risk factors may improve the identification of primary prevention subjects who may benefit from more intensive risk factor modification [32,33]. In this context, a large cohort study (330,201 patients from the UK Biobank) showed the predictive gain of a CAD PRS (241 genome-wide-significant single-nucleotide variations), especially in younger subjects, and its usefulness in identifying patients with borderline or intermediate clinical risk who might eventually benefit from early statin therapy [34]. PRS could represent one key element in a multifactorial assessment for risk stratification, including family and patient histories, clinical variables (e.g., key imaging biomarkers such as the coronary calcium score), lifestyle and environmental exposures, and other omics data (e.g., proteomics and metabolomics), to improve a personalized disease risk prediction assessment and allow early and increasingly targeted strategies to reduce the risk. In this context, very encouraging data have shown that subjects with a high PRS for CAD also showed a greater burden of subclinical atherosclerosis and benefited most from statin therapy to prevent their first coronary heart disease event [35].

However, as the predictive value of the PRS may greatly vary across different ethnic groups, and as the majority of studies were conducted in European cohorts, transferability to other populations is difficult [36]. In addition, the clinical efficacy and cost-effectiveness of some aspects need to be further investigated; e.g., their integration into existing clinical workflows to demonstrate incremental clinical benefits over established risk prediction models, their assessment in young people to estimate long-term risks, or the significance of cascade screening for family members of a proband with a high PRS [36]. Yet, the difficulty remains in how to prioritize which PRS to utilize to predict CV risk. Furthermore, beyond a lack of standardization regarding PRS construction, interpretation, and the harmonization of procedures, other questions of concern include how to overcome difficulties in current knowledge on how to appropriately prescribe, interpret, and communicate genetic results from health professionals to patients—all of whom have scarce familiarity with the concept of polygenic risk [37]. For these reasons, although current American Heart Association (AHA) and the European Society of Cardiology (ESC) documents acknowledge the potential of polygenic risk scores (PRSs) to refine cardiovascular risk stratification and support precision prevention, their routine implementation in clinical practice remains a long way off [38,39,40]. In particular, the AHA Scientific Statement (2026) highlights the need for further data in terms of clinical utility, cost-effectiveness, and applicability across different populations, given the underrepresentation of non-European populations in the datasets used to derive most currently available scores. whereas the 2025 ESC Clinical Consensus Statement provides a strategy for future implementation (e.g., application of the PRS in selected clinical scenarios) while emphasizing that the PRS is not yet recommended for routine cardiovascular risk assessments. 

### 2.3. Genes and Air Pollutants

Whereas epidemiological data account for a relationship between exposure to air pollutants and CVD, the importance of this association may be different across subjects, as air pollution interacts with genetic susceptibility. Mechanistic interaction includes several key connected molecular pathways involved in CV pathophysiology, including (a) thrombosis and coagulation, (b) oxidative stress pathways, (c) inflammatory signaling, (d) endothelial dysfunction, and (e) lipid metabolism. Nonetheless, other genes belonging to additive pathophysiological pathways may contribute to the effects of air pollution in determining different cardiovascular susceptibilities. In this regard, for example, the relationship between short-term PM2.5 exposure and blood pressure changes was modulated by variants in MMP1 and ITPR2 (renin-angiotensin-related genes) [41].

(a) Initial studies in 2009 evidenced how the FGB rs1800790 polymorphism of fibrinogen modified the fibrinogen response to PM10 in acute MI. It was also demonstrated that IL6 and fibrinogen gene variants modulated the blood IL-6 response to air pollution (CO, NO_2_, PM10, PM2.5, particle number concentration, air temperature, and relative humidity) in patients with a past history of MI, highlighting the importance of inflammatory processes in this relationship [42,43]. Since then, other studies have investigated the interaction between air pollution and candidate genes on CVD; essentially, the potential modification roles of genes related to inflammation (e.g., IL6, TNF) and coagulation (e.g., FGB, PAI-1) on the effects of short- and long-term exposure to air pollutants (including NO_2_, SO_2_, PM10, and O_3_) towards biomarker levels and the risk of cardiovascular adverse outcomes.
(b) Genetic variations in antioxidant enzymes, critical for antagonizing excess oxidants and preventing oxidative stress, are the most studied, in particular, genes belonging to the glutathione S-transferase (GST) family [44].

For example, the interaction between genes related to oxidative stress, GSTP1, GSTT1, and GSTCD, and long-term NO_2_ exposure on AMI risk was studied in 119 AMI cases and 1310 controls. The results suggested that pollution exposure was associated with an increased MI risk, and this risk varied according to genotype groups for variants in the GSTP1, GSTT1, and GSTCD genes (although it did not reach statistical significance [45]).

Several studies report how the GSTM1 null genotype may influence the interaction of air pollutant-induced CV changes affecting heart rate variability, endothelial dysfunction (e.g., brachial artery dilatation and soluble vascular cell adhesion molecule-sVCAM-1), and systemic inflammation [44].

Polymorphisms in several oxidative stress-related genes, such as GSTM1, GSTP1, GSTT1, HFE C282Y, CAT, *NQO1*, and *CYP1A1*, as well as heme oxygenase, have been found to be associated with vulnerability to air pollution-induced CV effects; however, for some of them, the available evidence is less clear [24].

Recent data have shown that PM2.5 induced the upregulation of HMOX1 expression, potentially indicating a self-defense response to protect endothelium by inhibiting HIF1α-mediated RAS activation [46].

Together, these results suggest that targeting endogenous protective pathways might be a possible strategy for protection from PM2.5-induced cardiovascular damage [25].

(c) Polymorphisms concerning inflammatory cytokines modulate responses to air pollution. In 1192 MI patients and 1506 controls, the interactions between variants related to inflammation (IL6 and TNF SNPs), traffic NO_2_, and heating SO_2_ emissions (up to 30 years retrospectively), and NO_2_, SO_2_, PM10, and O_3_ measurements used to estimate short-term (up to 5 days) air pollution exposure for AMI risk in relation to inflammatory biomarker levels were studied. The results demonstrated that genetic IL6 and TNF variants modulate long- and short-term pollution exposure effects on inflammatory marker levels, as well as MI risk [47]. Moreover, other genes involved in immune responses and inflammation could be implicated (e.g., transient receptor potential channel TRPA1 and TRPV1 and toll-like receptor TLR2 and TLR4 variants) [48].
(d) Regarding endothelial function, specific eNOS variants have been further investigated, suggesting that air pollutants can cause endothelial dysfunction and oxidative stress [49]. Moreover, a meta-analysis demonstrated an increased risk of CVD in subjects carrying the NOS3 894 TT polymorphism in association with air pollution exposure [50].
(e) Some gene polymorphisms (apolipoprotein E (APOE), lipoprotein lipase, and vascular endothelial growth factor (VEGF) genes) were found to be related to lipid metabolism and endothelial function and to modify the association between PM2.5 and heart rate variability [51]. APOA5 1131 is one of the most studied polymorphisms in association with CVD for its role in epigenetic pathways [52]. PCSK9 also emerges as an interesting candidate gene, although evidence still remains insufficient [53].

### 2.4. Gene–Air Pollution Interaction on CV Risk

The majority of previous studies that evaluate interactions between different types and levels of air pollutants and genes have essentially focused on the candidate gene approach (mostly related to detoxification and inflammation), with small sample sizes (e.g., 12 SNPs; a fact that may reduce the power to evidence significant associations with disease outcomes and increase heterogeneity between studies) [54,55]. Clearly, gene–air pollution interactions may affect many different cellular pathways, altering the concentration of circulating factors, which then may drive the onset and growth of adverse disease events. In this context, some studies have focused on the causality of particulate matter on cardiovascular disease, exploring how genetic variants, mainly related to inflammation and oxidative stress processes (most studied loci are IL-6, TNF-α, GSTM1, and the fibrinogen gene cluster), modified the associations between blood CV risk biomarkers (e.g., homocysteine, C-reactive protein, or cytokines) and exposure to pollutants (e.g., PM2.5, PM10, O_2_, and SO_2_) [56]. Thus, the study of gene–environment interactions may be helpful for identifying subjects at higher CV risk, improving disease prediction, and providing further knowledge of biological events. In this context, a recent study, which aimed to evaluate the causality of PM on CVD and cardiovascular biomarkers by using a Mendelian randomization (MR) analysis, identified 16 single nucleotide polymorphisms (SNPs) for PM2.5 and 6 SNPs for PM10 from UK Biobank participants. The results suggested a causality of PM2.5 with a higher risk of MI and triglycerides and decreased HDL-C levels, as well as a causality of PM10 with a risk of heart failure, ischemic stroke, and reduced HDL-C [57]. 

Recently, the interaction of genetic risk (based on 540 genetic variants) with air pollution exposure (long-term PM2.5 exposure assessed using satellite-based PM2.5 estimations at a 1 km resolution) on CAD risk was evaluated in 41,149 subjects from the Prediction for Atherosclerotic Cardiovascular Disease Risk in China (China-PAR) project. These subjects were followed for a median of 13.01 years of follow-up, during which 1373 incident CAD events were observed. While long-term PM2.5 exposure and high genetic risk individually significantly increased CAD risk, a significant multiplicative (*p* < 0.001) and additive interaction between genetic risk and PM2.5 exposure on CAD risk was also observed [58]. Another study evaluated, beyond the separate effects, the gene–air pollution interaction on CAD risk by using a PRS and Cox proportional hazard models in the UK Biobank (487,507 participants who were free of CAD at baseline and followed for a median of 8.8 years). Both a high PRS (40 SNPs) and air pollution exposure (PM2.5, PM2.5−10, PM10, NO_2_, and NOx) conferred a higher CAD risk. Moreover, an additive effect for air pollution and genetic factors on the development of CAD was observed [9].

A total of 249,082 white British participants without overt CVD from the UK Biobank cohort (aged 56.9 ± 8.0 years, 46.8% men), followed for a median of 10.8 years, were studied to assess the combined effect of air pollution (PM10 or PM2.5) and genetic susceptibility (by using the PRScs method, which integrates the effect size of millions of single variants, reflecting the linkage disequilibrium structures of the target population) on CV risk (all-cause and cardiovascular mortality, coronary artery disease, MI, stroke, ischemic stroke, heart failure, and atrial fibrillation). The results showed that the combination of higher exposure to PM2.5, together with a high genetic risk, was associated with a higher incidence of outcomes. However, no significant interactions were observed between genetic risk and PM2.5 on CV death or CVD events [10]. Moreover, the effects of genetic susceptibility on the interaction between pollution and CVD were studied, employing data from about 300,000 UK Biobank participants on 10,745,802 variants [10]. Interestingly, the genome-wide interaction analysis identified 4, 13, and 32 SNPs interacting with PM2.5 in its association with CAD, ischemic stroke, and peripheral artery disease, respectively; gene-set enrichment analysis involved pathways related to cell–cell adhesion, deoxyribonucleotide biosynthesis, RNA metabolism, and calcium ion homeostasis [59]. Moreover, animal studies further strengthened the role of PM2.5 exposure in interacting with adenosine kinase expression (ADK) to affect the onset and progression of atherosclerotic lesions, suggesting that ADK is, therefore, a potential susceptibility factor and could otherwise act as a promising therapeutic target to counteract PM2.5-induced atherosclerosis [59].

There are also some data regarding MI; a study based on 456,354 UK Biobank participants, evaluating annual mean pollutants (PM2.5, PM10, NO_2_, and NOx), evidenced that subjects with both a high PRS and high pollution exposure showed the greatest MI risk among all participants (∼255% to 324%) [60].

Moreover, when data from 329 189 UK Biobank participants without an MI at baseline were analyzed, the results suggested that integration of a PRS (38 SNPs), air pollution exposure, and traditional lifestyle and clinical factors may improve the risk prediction of MI, including its two subtypes (STEMI and NSTEMI) [61]. The addition of a PRS to the use of traditional clinical risk tools for different complex diseases (including cardiometabolic diseases) showed that a PRS may provide additional value in clinical risk prediction, especially for HF and early-onset cases, which underlie the PRS’s possible key contribution to preventive strategies for susceptible high-risk subjects in this clinical setting [62]. Given its potential value, the PRS is also under careful scrutiny for integration into primary prevention of cardiometabolic diseases, where it may help develop a proactive approach to clinical disease management that could refine patient risk and promote the development of personalized medicine [63]. Studies are also now being conducted to assess the PRS’s clinical utility in real life; a recent study evidenced that absolute CVD risk (based on a clinical risk score) and relative genetic risk (based on a PRS) provide independent information and increase accuracy in predicting incident CVD [64]. Interestingly, a recent study reported how an integrated genomic model, which integrated different CAD risk-related determinants (polygenic risk scores, genetically proxied proteomic and metabolomic risk scores, and clonal hematopoiesis of indeterminate potential) was able to represent their cumulative effects in patient risk stratification (e.g., helping to identify subjects at high risk despite not carrying any single major variant, as well as those at low risk even when carrying known high-risk genetic factors) and improve the predictive capacity of available clinical tools, such as traditional cardiovascular risk calculators [65]. Thus, although incremental gains are limited in the general population, the use of these comprehensive genomic models, integrating different biological markers, opens the possibility of an earlier and more accurate refinement of CVD risk in the future in specific patient populations.

The concept of environmental exposome is an important emerging aspect of this field, which identifies air pollutants as key inducers of epigenetic modifications that may modulate cardiovascular risk independently of DNA sequence variations, including DNA methylation, histone modifications, chromatin remodeling, and variations in microRNA and long non-coding RNA expression [66].

A very recent review explores this topic in depth, discussing available data on air pollution and CVD-associated epigenetic changes [67].

**Figure 1:**
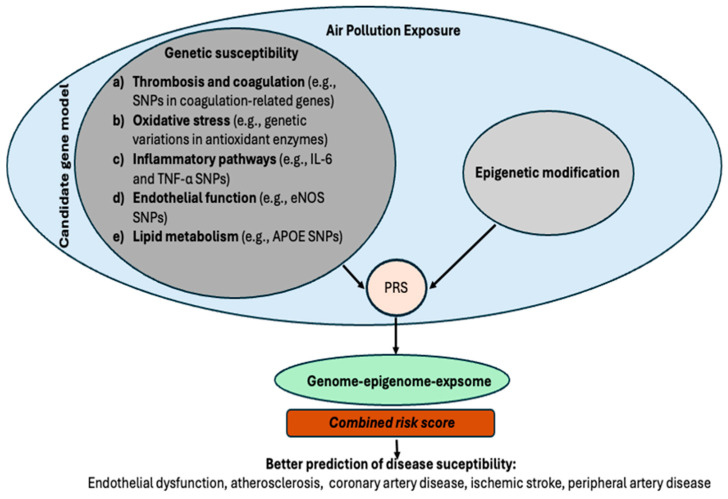
A candidate gene model and the study of epigenetic modification: passage to the integration of polygenic risk scores (PRSs, static inherited risk), epigenome (dynamic inherited risk), and exposome (environmental effect on cardiovascular disease-CVD) allows the creation of combined risk scores to better predict cardiovascular (CV) susceptibility. SNPs: single nucleotide polymorphisms; IL-6: Interleukine 6; TNF-〈: Tumor necrosis factor alpha; eNOS: endothelial nitric oxide synthase; APOE: apolipoprotein E.

Interestingly, epigenetic changes emerge as modulators of the functional effects of inherited polymorphisms, opening the possibility of a combined genome–epigenome–exposome scenario, allowing for the replacement of current research based on the candidate-gene model and representing future research strategies in the field of precision cardiovascular medicine (Figure 1).

## 3. What Remains to Be Better Studied

### 3.1. Limitations in the Assessment of Air Pollution Risk

Air pollutants are complex mixtures containing various gases, liquids, and particulates (including PM2.5, PM10, O_3_, NO_2_, and NOx), whose physical and chemical complexities and the variety of biological responses elicited render it hard to evaluate the significance of the relationship between this variety of pollutants and diseases. Thus, wide differences in the chemical and physical properties (mass, number, size, shape, surface area, reactivity, acidity, solubility, and the internal or surface positioning of chemicals on the particles), composition (metals, salts, organic particles, or biological substances), variability of formation processes of particles (nucleation, condensation, coagulation, mechanical processes), as well as differences between anthropogenic and natural emission origins (traffic, industrial activities, biomass burning, mineral desert dust, sea spray, biogenic emissions) multiply the variety of conditions to an extreme extent [25].

The characteristics of the geographical area in which exposure is assessed are another key parameter of variability. Moreover, the possibility of a different relationship between exposure and genetic risk for CAD in highly polluted areas, where generally studies are planned, vs. zones with relatively low environmental conditions, requires further consideration in future studies. Similarly, studies distinguish between “short-term” and “long-term” effects, where short-term exposure may destabilize susceptible plaques and precipitate acute CV events, whereas long-term effects may support the onset and development of cardiometabolic risk factors (e.g., hypertension, type 2 diabetes, endothelial dysfunction), ultimately favoring dyslipidemia and atherosclerotic evolution. Of course, this distinction is only theoretical, as both effects contribute to the same final overall adverse effects.

One of the main limitations of these types of studies is that the specific real-time environmental exposure of each subject cannot be punctually measured; instead, exposure is generally estimated in relation to residential addresses, without considering individual variations in activity levels, occupational exposure, and indoor exposure, all factors that may potentially be a cause of exposure misclassification. Differences between fixed monitoring points and mobile or individual monitoring assessment (e.g., wearable devices, sensors mounted on vehicles, geospatial assessments, fixed monitoring stations) and the use of monitoring instruments with different spatial and temporal resolutions may also greatly affect the final results [25].

This is further complicated by the fact that, despite adjustments for major pollutant confounders, unknown or unmeasured additive confounding parameters might interact and influence the risk of disease and outcomes. In this context, the burden of other environmental components (beyond PM2.5, the less studied NO_2_, NOx, O_3_, and SO_2_, and volatile organic compounds, whose effects on health are not clearly established currently) and other meteorological parameters (e.g., temperature, humidity, barometric pressure, and wind speed, which are often neglected) and their interactions need to be further explored. 

### 3.2. Limitations in the Assessment of the Genetic Risk

At present, the majority of published studies have still been performed using a candidate gene approach, which may greatly affect the estimation of the genetic effect; this fact demonstrates the need for further genome-wide studies, which may allow for the discovery of new interactions. Moreover, the majority of available data are obtained from Caucasian cohorts, limiting the generalizability to other populations and raising the need for studies to be conducted across different ethnic groups. Furthermore, the effect of sex also requires further exploration. Another limit is that the possibility of having independent replication is challenging because interaction studies never focus on the same exposure or outcome used in other trials, a fact that is important when demonstrating that the interactions found are not cohort-specific. Power also remains one of the most frequent and important obstacles to overcome in gene–environment interaction studies. Although efforts are being made to develop more efficient and advanced statistical analyses, other key factors require improvement, such as increasing the sample size, incorporating additional exposures into analyses, improving exposure assessment, and controlling for confounding factors [68]. In fact, correlations between environmental exposures and other parameters may complicate the interpretation of results. Thus, other variables (such as diet, physical activity, smoking habits, and socio-economic variables), which may be crucial in disease progression and the modulation of gene susceptibility, need to be collected and integrated into the models to improve predictability. However, the use of self-reported data for these covariates might reduce the power of the associations, introducing errors due to misclassification. Then, even once a significant gene–environment interaction is found, the clinical relevance of this interaction may be difficult to interpret and communicate. Accordingly, once the genetic risk loci are identified, additional in vivo and in vitro studies may be further required to fully interpret the interactions and establish the causative molecular mechanisms underlying disease, as well as for functional validation. In addition, these studies should introduce biomarker measurements that are followed over time so as to better understand the functional significance of these variants and how they can affect disease trajectories, holding mechanistic roles in the interaction with environmental pollutants. The best time to perform such tests is also still indefinite, and disclosure of genetic risks is challenging.

In this context, AI-driven computational predictive models for CV risk prediction that integrate the PRS, environmental, clinical, imaging, biochemical, and lifestyle factors may improve discrimination of risk over traditional risk calculators, providing further insights into disease development and therapeutic responses, and allowing precision medical approaches [69,70].

Along this line of reasoning, a model developed to select a group of candidate parameters for CAD risk prediction using machine learning, including a PRS beyond anthropometric and social risk factors, and using hematological data of 173,274 UK Biobank subjects, improved discrimination of CAD risk when compared with the Framingham risk score, pooled cohort equations, and QRISK3 [71]. Similarly, application of machine learning and deep learning models to 10-year CVD event prediction by using longitudinal electronic health records (anthropometric, laboratory, and clinical data) and genetic data from 109,490 subjects improved predictivity compared to the tools currently utilized in routine clinical practice (e.g., the American College of Cardiology and the American Heart Association (ACC/AHA) Pooled Cohort Risk Equation) [72]. Moreover, in 95,935 individuals, an AUC of 0.95 for CAD risk prediction was achieved with the use of electronic health record-based machine learning that integrates traditional risk factors (anthropometric, laboratory, and clinical data), pooled cohort equations, and PRSs. In addition, the model accurately quantified the degree of coronary stenosis, complexity of disease, and risk of death, identifying a subset of underdiagnosed patients [73].

Not least, despite undoubted advancements, other aspects need to be addressed in order to warrant effective and ethical application in healthcare, especially regarding data privacy, algorithm bias, and AI model standardization. The main limitations in the assessment of air pollution/genetic risks are summarized in Table 2.

## 4. Conclusions

There is substantial evidence that associations between air pollution exposure (over both short- and long-term periods) and adverse CV outcomes are modulated by genetic variants, especially, but not only, those in genes that are related to detoxification and inflammation. Many limitations are not negligible and require considerable efforts, both in terms of pollution monitoring and in elucidating the role of genetic risk factors in the atherosclerotic process, as well as refinement in terms of analytic and clinical validity and evaluation of real clinical advantages.

As such, this research area can provide further knowledge of the etiology of CVD, offering new tools for targeted prevention and treatment for more susceptible patients, towards a more personalized medical approach. In fact, while improving air quality, in general, can benefit the entire population, individualized interventions to alleviate the effects of air pollution exposure in those at high genetic risk may be valuable for more vulnerable subsets of the population.

## Figures and Tables

**Table 1:** Number of participants, pollutants considered and associated events in the study cited in Section 2.1.

Number of Cases Evaluated	Air Pollutants Exposition	Main Results	Ref
7,300,591 subjects	PM2.5	Increase in all-cause mortality, cardiovascular disease, cardiovascular disease mortality	[12]
6,336,506 subjects	PM10	Increased risk of MI	[13]
>65 million participants	O_3_	Increased risk of hospital admissions for MI and stroke	[18]
18,035,408 cases of ischemic stroke	NO_2_, O_3_, CO, SO_2_, PM2.5, and PM10	Increased incidence of ischemic stroke and stroke mortality	[19]
7,568,876 heart failure events	PM2.5, PM10, NO_2_, SO_2_, CO, and O_3_	Increased heart failure	[17]
663,276 angina pectoris events	PM2.5, PM10, NO_2_, and CO	Increased risk of angina	[20]
28,186,905 participants	PM2.5, PM10, NO_2_, SO_2_, CO, and O_3_	Increased risk of hospitalization due to ischemic heart disease	[21]
11,656 participants	NO_2_ and PM10	Increased insulin resistance	[23]

**Table 2:** Limitations in the assessment of air pollution/genetic risks.

**Pitfalls in the assessment of air pollution risk** (1) **More data on O_3_, NO_2_, PM10, volatile organic compounds** - **Particles: physical structure, chemical composition, mechanism of formation** - **Differences between anthropogenic and natural emission origins** (2) **Characteristics of the geographical area where the measurements are conducted** (3) **Risk of exposure misclassification** (4) **Differences due to fixed monitoring points and mobile or individual monitoring assessments**	**Pitfalls in the assessment of genetic risks** (1) **A need for genome-wide studies** (2) **Effects of sex** (3) **Effects of race** (4) **Lack of generalization due to cohort specificity** (5) **Power of the study** (6) **Integration with other significant variables of a different nature** (7) **Limitation of self-reported data** (8) **Age to perform tests** (9) **Other biomarker measurements over time** (10) **Ethical issues**

## Data Availability

No new data were created or analyzed in this study. Data sharing is not applicable to this article.
